# Optimization and Validation of Arabinoxylan Quantification in Gluten-Free Cereals via HPAEC-PAD Based on Design of Experiments

**DOI:** 10.1021/acs.jafc.5c02445

**Published:** 2025-04-02

**Authors:** Katharina Hoefler, Ulrich Sukop, Stefan Scheler, Elisabeth Reiter, Denisse Bender, Mario Jekle, Regine Schoenlechner, Stefano D’Amico

**Affiliations:** † AGES − Austrian Agency for Health and Food Safety, Institute for Animal Nutrition and Feed, Spargelfeldstraße 191, 1220 Vienna, Austria; ‡ BOKU − University, Department of Biotechnology and Food Science, Muthgasse 18, 1190 Vienna, Austria; § University of Hohenheim − Department of Plant-Based Foods, Garbenstraße 25, 70599 Stuttgart, Germany; ∥ University of Applied Sciences Kaiserslautern − Department of Applied Logistics and Polymer Sciences, Carl-Schurz-Straße 10 − 16, 66953 Pirmasens, Germany

**Keywords:** maize, oat, rice, acid hydrolysis, monosaccharide analysis, response surface methodology

## Abstract

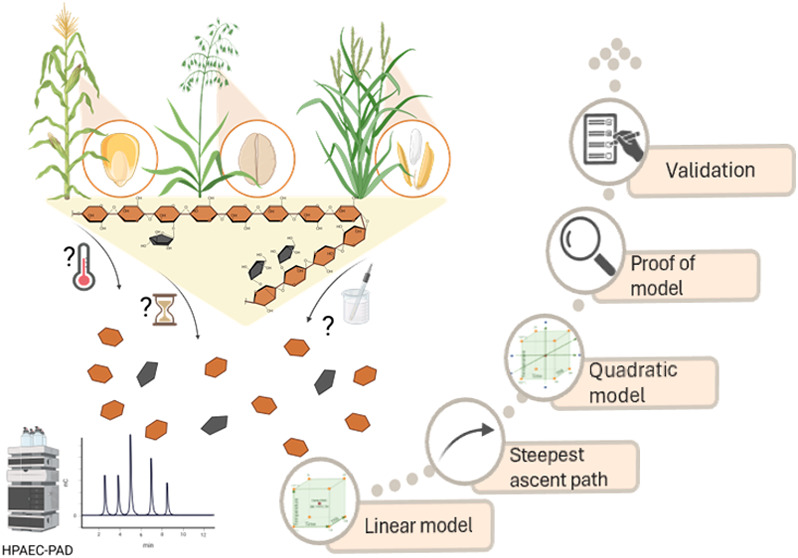

Arabinoxylans (AXs) are dietary fibers in monocotyledon cell walls that benefit digestive health and enhance food functionality. Despite their importance, no standardized method exists for AX quantification in gluten-free (GF) cereals. This study investigated the effect of hydrolysis parameters for AX quantification in GF cereals (maize, rice, oat) with varying AX content and nutritional profiles to address matrix effects. The effects of trifluoroacetic acid (TFA) concentration (0.25–4 M), temperature (90–127 °C), and time (1–5 h) on hydrolysis efficiency were examined, whereby temperature showed, in contrast to acid concentration and time, a pronounced influence. The design of experiment (DoE) model predicted 2 M TFA, 2.4 h, and 103 °C as the optimal conditions for maximizing AX yield without detectable monosaccharide decomposition for all varieties. This was experimentally confirmed with a deviation of less than 10%. An extensive validation confirmed the method’s accuracy and reliability for this unified method.

## 1 Introduction

Arabinoxylans (AXs) are the major hemicellulose polysaccharides that occur in the cell walls of cereals.^1−3^ The general structure of AXs is consistent throughout all cereals, comprising a linear ß-1-4 xylopyranose backbone with side chains of α-arabinofuranosyl linked to the xylose units at position O-2 and/or O-3.^4−6^ However, there are noteworthy differences in the fine structure, quantity, and localization of AXs in various cereal species.^7^ The arabinose to xylose (A/X) ratio is typically used for a first characterization as it is decisive for the solubility of AXs, besides the degree of polymerization. But the distribution of the arabinose side chains varies considerably between different cereal species, resulting in AXs displaying distinct properties. Furthermore, the content and A/X ratio also vary depending on the specific location within the kernel.^3,8^ Typically, the outer layers of the kernel exhibit higher AX content and a higher A/X ratio.^8,9^ Additionally, minor amounts of other sugar residues, including galactopyranose or glucuronic acid, and hydroxycinnamic acids such as ferulic acid (FA), esterified at the O-5 position of the arabinose, can be present, leading to complex and highly varying AX structures.^4,6,9,10^ These heterogeneous structural composition of AXs consequently generates a diversity of physicochemical and functional properties.^7,11^

Wheat has been the predominant source of AX, but other sources, such as maize AX, are becoming increasingly relevant due to their possible recovery from industrial byproducts, and their potential to provide customized improvements.^11,12^ A notable potential application of AX is in the production of gluten-free (GF) foods, where incorporation of AX can improve flavor, nutritional value, and physical product quality.^13^ However, for the future use of AX in these sectors, it is required to overcome solubility issues of byproduct-derived AX and a reliable method to analyze AXs in different cereal products, which is a complex and challenging task, as the way of calculating the AX content, the methods of detection, and already the hydrolysis itself vary considerably within the applied methods to date.

The process for quantifying AX involves the release of the maximum amount of monosaccharides through hydrolysis of glycosidic bonds, followed by their subsequent quantification.^14^ The hydrolysis can be conducted either through enzymatic or acid hydrolysis or by methanolysis.^14^ Currently, the predominant hydrolysis process for the quantification of monosaccharides is acid hydrolysis, which primarily employs the use of H_2_SO_4_, HCl, or trifluoroacetic acid (TFA).^14^ TFA has the advantage that it can be easily evaporated, thus obviating the necessity for a neutralization step and causing less damage to the monosaccharides, which is of major concern, because both, xylose and arabinose, can undergo degradation to 2-furaldehyde and further to formic acid, resulting in an underestimation of the monosaccharide content.^15,17^ The release of monosaccharides is dependent on the structural attributes of the polysaccharide itself, as well as stability of the respective monosaccharide.^16,17^ This has led to a wide range of hydrolysis methods being employed to achieve a balance between maximum release and minimum degradation.^14^ For example, the TFA hydrolysis conditions currently employed range from 1 to 4 M at temperatures between 80 and 135 °C for periods between 1 and 12 h.^14,18−23^

Calculation of the final AX content is also not carried out uniformly. The different variations include the summation of arabinose and xylose, the inclusion of a correction factor of 0.88 due to the abstraction of water during the condensation reaction, as well as the additional deduction of the arabinose content released from other fibers e.g., arabinogalactans.^24,25^ Furthermore, the degradation products such as 2-furaldehyde can be included, which complicates the AX determination additionally.^26^ One further challenge in developing standardized methods for AX determination is the lack of certified reference materials (CRMs). Thus, the overall objective of this study was to develop a reliable and easy method to determine the AX content across different matrices. Therefore, GF grains (maize, oat, and rice), which strongly differ in their composition, especially in concentration, type, and structure of dietary fiber, were selected. For each variety, the three key parameters of the hydrolysis, TFA acidity, hydrolysis time, and temperature, were optimized via response surface design. Subsequently, the method was extensively validated, including both sample preparation and detection via high-performance anion exchange chromatography with pulsed amperometric detection (HPAEC-PAD), to prove the reliability and robustness of the entire determination process.

## 2 Materials and Methods

### 2.1 Materials

The oat, maize, and rice cereal samples used in this study were *Avena nuda* L. (cultivar: Talkunar) harvested in 2022 in Gusbor, Lower Saxony, Germany (sample code: O01), *Zea mays* L. (cultivar: Santana (DKC3972)) harvested in 2021 in Neudorf bei Staatz, Lower Austria, Austria (sample code: M01), and *Oryza sativa* L. ssp. *Japonica* (cultivar: Carnoli Classico) harvested in 2022 in Provinz Vercelli, Italy (sample code: R01). Sodium hydroxide solution (49–51%) in ion chromatography (IC) quality (as HPAEC eluent) and the sugar calibration standards xylose, arabinose, galactose, fructose, rhamnose, glucose and mannose, ribose, and melibiose with a purity of at least >98% as well as TFA for synthesis were purchased from Sigma-Aldrich (St. Louis, Missouri, USA) and high- and medium-viscosity wheat AX (P-WAXYM and P-WAXYH) from Megazyme (Wicklow, Ireland) with a purity of >95%. The GF wheat starch SANOSTAR was purchased from Kröner GmbH (Ibbenbüren, Germany). Ultrapure (UHQ) water was used for all analyses and was provided by ultrapure water systems. All other solvents and reagents, unless otherwise stated, were of analytical grade or superior quality.

### 2.2 Chemical Analysis

Prior to analyses, the grains were ground with an ultracentrifugal mill Retsch ZM 200 (Retsch, Haan, Germany) equipped with a 500 μm sieve and stored at 2–8 °C in sealed plastic containers. The dry matter content was calculated by determining the weight loss of a sample quantity of 10 ± 1.00 g after drying for 1 h at 130 ± 2 °C using an automatic Brabender moisture analyzer type MT-C (Anton Paar, Graz, Austria). The crude protein content was determined via the Dumas combustion method using a DuMaster D-480 (Büchi Labortechnik AG, Flawil, Switzerland) with the calculation factor 6.25 for maize, 5.5 for oat, and 5.95 for rice while using aspartic acid for calibration according to ISO 16634-1:2008. The total starch content was determined using the total starch (AA/AMG) assay kit (K-TSTA) from Megazyme Ltd. (Wicklow, Ireland) in accordance with AACC 76-13.01. The crude fat content was measured via Soxhlet extraction by using a Hydrotherm HT V (C. Gerhardt GmbH, Königwinter, Germany) according to ICC 136 and ash content was determined gravimetrically by using a Carbolite Gero AAF 1100 oven (Verder Scientific GmbH, Haan, Germany) according to ICC 104. The TDF content was determined using the total dietary fiber assay kit (K-TDFR) Megazyme Ltd. (Wicklow, Ireland) in accordance with AACC 32-07.01. The total phenolic acid content (TPC) was determined via Folin-Ciocâlteu reagent based on the method by Speranza et al. with slight adaptions of sample weight (500 mg) and reagent volume during extraction of bound phenolic acids (3 mL of 80% ethanol, 2.1 mL of 2 M sodium hydroxide, 450 μL of 12 M hydrochloric acid and 1.4 mL ethyl acetate).^27^ The amino acid composition was determined by using a biochrom 30Plus Amino Acid Analyzer (Biochrom Ltd., Cambridge, U.K.) according to VO (EU) 152/2009 Annex III.E, as amended by regulation (EU) 2024/771.

### 2.3 Acid Hydrolysis of Arabinoxylans

For sample preparation, 200 ± 10 mg of sample was weighed into 50 mL polypropylene centrifuge tubes, suspended in 5 mL TFA in varying concentrations (0.25–4 M), and vortexed (1 min). The samples were hydrolyzed in a Durocell Eco line oven (MMM Group, Munich, Germany) at temperatures ranging from 90 to 127 ± 2 °C. During hydrolysis, the samples were gently shaken every 30 min. The total duration of the hydrolyzation ranged from 1 to 5 h. To stop the hydrolyzation, the samples were cooled down by the addition of approximately 50 mL of cold UHQ water and 50 μL Carrez I (15% potassium hexacyanoferrate solution) and 50 μL Carrez II (30% zinc sulfate solution) to precipitate nonhydrolyzed macromolecules. The samples were centrifuged (10 min, 2000 *g*, 20 °C), then 0.5 mL aliquots from the supernatant were transferred into Eppendorf tubes and vacuum-dried (45 °C, 3 h, <2000 Pa) using a Savant SpeedVac SPD1030 vacuum concentrator (Thermo Fisher Scientific, Sunnyvale, CA, USA). The dried samples were stored at room temperature prior to HPAEC-PAD analyses.

### 2.4 Monosaccharide Quantification via HPAEC-PAD

The dried samples were resuspended in 1 mL of UHQ water and vortexed for 1 min, filtered (0.25 μm nylon filter), and diluted before injection into the chromatography system. A Dionex ICS-6000 DC HPAEC-PAD system (Thermo Fisher Scientific_,_ Sunnyvale, CA, USA) equipped with a CarboPac PA20-Fast (2 × 100 mm, 4 μm) and corresponding guard column was used. A column temperature of 30 °C with gradient elution (conditions listed in Table S1) at a flow rate of 0.2 mL/min was applied. Quantification was performed by external calibration (0.1 to 20 mg/L) using multicomponent standard solutions of fucose, arabinose, galactose, glucose, xylose, mannose, and fructose. Peak areas were integrated using Chromeleon 7 Chromatography Data system version 7.2.10 (Thermo Fisher Scientific Sunnyvale, CA, USA) and the amount of AX was calculated as the sum of arabinose and xylose.

### 2.5 Experimental Design and Data Analysis

Response surface methodology (RSM) was used to optimize the hydrolysis conditions for AX quantification in terms of hydrolysis time (2–4 h), TFA concentration (0.25–4 M), and temperature (90–127 °C). For each matrix (M01, O01, and R01) a 2^3^ full factorial experimental design (FFD) was performed independently to create a linear model. From these results, the path of the steepest ascent was calculated for each matrix to find the area of maximum response. Subsequently, a central composite design (CCD) was created at the optimum region to fit a second-order response surface. Finally, the model was confirmed by experimental data. The models were analyzed with the software *R* (version 4.2.1 “Funny-Looking Kid”) using the *rsm* package (version 2.10.4).^28,29^ The quality of the regression models was assessed by the coefficient of determination *R*^2^, and the statistical significance was analyzed using analysis of variance (ANOVA) at *p* < 0.05.

For the FFD, four replicates of the central point (110 °C, 2 M, 3 h) and eight single-run factorial points (±1 h, ±1 M, ±10 °C) were created independently for all three matrices. The central point was chosen based on the optimal hydrolysis conditions found by Uhliariková et al.^20^ The linear regression model was calculated based on eq 1, where *Y* is the predicted response expressed as relative AX content, the terms β_0_, β_*i*_, and β_*i,j*_ are the regression coefficients of the intercept, linear and two-way interaction parts, respectively, and *X*_*i*_ and *X*_*j*_ refer to the coded predictor variables, with *n* indicating the number of independent factors.
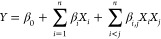
1

The rotatable CCD was created for each matrix with three replicates at the central point (level 0), eight factorial points as a single run (level 0 ± 1), and six axial points as a single run (level 0 ± α), whereby α was calculated as 1.73 according to Myers et al.^30^ The results were fitted to the regression model given in eq 2, where *Y* is the predicted response expressed as relative AX content, the terms β_0_, β_*i*_, β_*i,j*_, and β_*ii*_ are the regression coefficients of the intercept, linear-, two-way interaction (TWI) and quadratic part (PQ), respectively, and *X*_*i*_ and *X*_*j*_ refer to the coded predictor variables, with *n* indicating the number of independent factors.
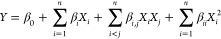
2

The predicted maximum response from the regression model was confirmed by performing triplicate experiments for each matrix and calculating the coefficient of variation (CV) of the predicted response from the obtained response. Further, the degree of hydrolysis was proven by evaluating the presence of oligomers from AX (AXO) and starch (SO). In detail, HPAEC-PAD chromatograms for M01, O01, and R01 at the calculated optimum hydrolysis conditions were compared to those from incomplete and fully hydrolyzed P-WAXYM and starch matrix.

### 2.6 Validation

For validation, the optimized hydrolysis conditions of 2 M TFA, 2.4 h at 103 °C were assessed for M01, O01, and R01. The method was validated with regard to trueness, precision, limit of detection (LOD), limit of quantification (LOQ), working range, and selectivity according to Eurachem validation guidelines.^31^ A minimum of three replicates were performed in all validation experiments. The GF wheat starch SANOSTAR, with an examined arabinose and xylose content < LOQ, was considered as a similar analyte-free matrix. For recovery experiments, this wheat starch was spiked with AX (1, 5, or 10% P-WAXYH, respectively). Peak area was integrated using Chromeleon software. The validation results pertaining to the AX content were calculated in accordance with the methodology proposed by Houben et al., whereby the sum of arabinose and xylose was multiplied by 0.88.^24^

#### 2.6.1 Instrument- and Method Working Range

To assess the instrument working range, an external calibration was performed with six different concentrations (0.1, 0.5, 1, 5, 10, and 20 μg/mL) in triplicate, and a calibration curve was fitted by quadratic regression. The method working range was calculated based on the instrument working range by including the dilution steps during sample preparation.

#### 2.6.2 Trueness

The trueness was assessed first by recovery experiments, whereby the GF wheat starch matrix was spiked with 1, 5, and 10% P-WAXYH and measured on the same day with four replicates each and expressed as percentage recovery. To assess possible degradation of the monosaccharides during the hydrolysis process, 20 ± 1 mg of each monosaccharide calibration standard was hydrolyzed. To evaluate the complete release of the monosaccharides, 50 ± 2 mg of P-WAXYM and P-WAXYH were used. The monosaccharide degradation measurements as well as the RM measurements were performed in triplicates within 1 day and expressed as percentage recovery.

#### 2.6.3 Precision

Precision was assessed by both repeatability (intraday precision) and interday precision. For repeatability, ten samples of O01, M01, and R01 were hydrolyzed on the same day and expressed as CV, respectively. For interday precision, triplicates of O01, M01, and R01 were measured on three different days and expressed as CV, respectively.

#### 2.6.4 Limit of Detection and Limit of Quantification

To obtain reliable LOD and LOQ values, including sample preparation steps, a small amount of analyte was spiked into a large amount of starch. Therefore, GF wheat starch with 1% P-WAXYH was used as a model with a high glucose-to-AX ratio, which is commonly found in cereals, to determine the LOD and LOQ. The sample preparation was performed ten times, and LOD and LOQ were then calculated using the standard deviation (SD) from ten measurements and the number of routine measurements (*n* = 2) according to eqs 3 and 4.
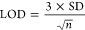
3
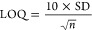
4

#### 2.6.5 Instrument Selectivity

The selectivity of the instrument was assessed by the simultaneous identification of the different monosaccharide calibration standards arabinose, galactose, glucose, xylose, mannose, fructose, ribose, and the disaccharide calibration standard melibiose at the same concentration level of 0.5 μg/mL diluted in UHQ water.

## 3 Results and Discussion

### 3.1 Compositional Analysis of Raw Material

**Figure 1:**
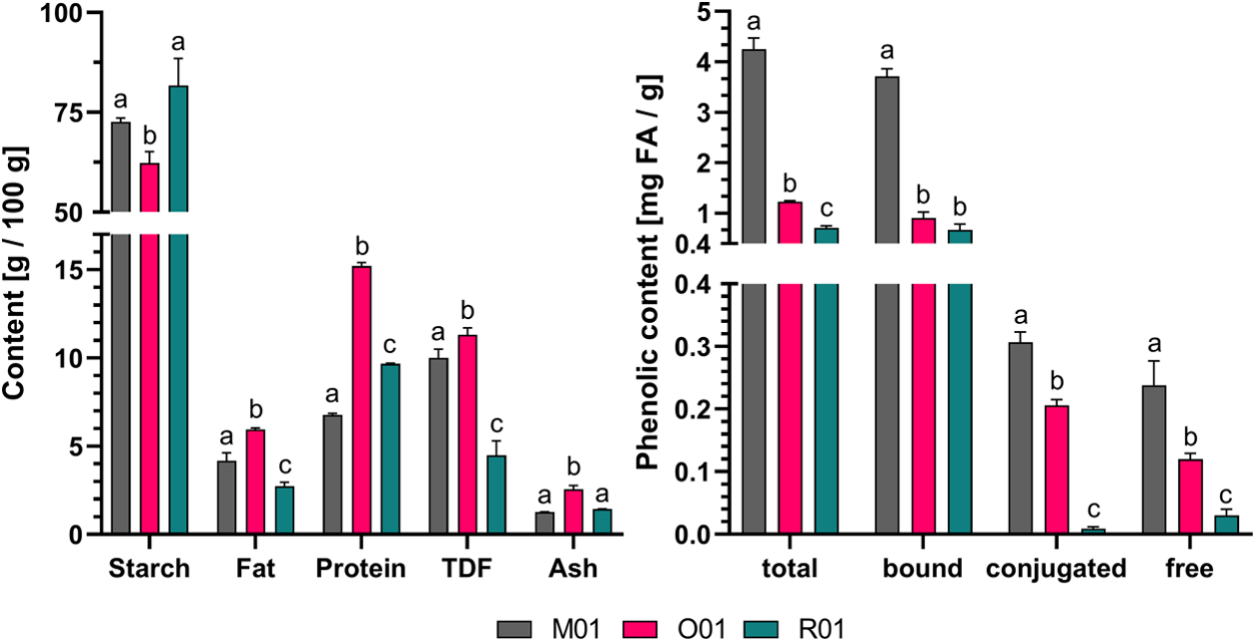
Composition of the three selected cereals, maize (M01), oat (O01), and rice (R01). The data are presented as mean + SD (*n* = 3) in dry matter with the lowercase letters indicating significant differences (*p* ≤ 0.05).

The chemical composition of the selected cereals for this study is shown in Figure 1 with the detailed values and amino acid profile given in the supplementary data (cf. Table S2 and Figure S1). In brief, oat exhibited significantly lower starch but higher fat, protein, TDF, and ash content than rice and maize. Rice was characterized by a significantly lower fat, protein, TDF, and phenolic content, whereby maize showed the significantly highest amount of phenolic acids. Thus, a distinct variability of the composition was covered for the selected samples, which facilitates the evaluation of potential matrix effects on the release or degradation of AX during the subsequent hydrolysis process. Additionally, the effect of differing AX structures (varying degrees of cross-linking with other ingredients such as lignin or proteins through FA cross-links) on the hydrolysis process was included. These structures can e.g., change the binding to the matrix and may influence the release in a hydrolysis process.^6,32^ In earlier studies, it has been shown that the A/X ratio has an influence on the solubility influencing the complex hydrolysis process of these heterogeneous polymers.^2,33^ Based on these results, the selected cereals provide an appropriate basis for investigating whether different GF cereals require differing conditions for optimal AX hydrolysis or if there is a single, universally applicable method whereby both the matrix composition and the AX type were considered.

### 3.2 Optimization of Acid Hydrolysis via DoE

#### 3.2.1 2^3^ Full Factorial Design (FFD) and Path of the Steepest Ascent

Initially, a FFD with four central points and eight factorial points was created for the three variables, TFA concentration, hydrolyzation time, and heating temperature for R01, M01, and O01 independently as single runs. The summarized results of the 2^3^ FFD, i.e., the effects of TFA, hydrolyzation time, and heating temperature on the AX content (g/100 g) are given in Table 1, and the corresponding monosaccharide values of arabinose, xylose, and galactose are listed in Tables S5–S8 (run nos. 1–12). Based on these results, a linear regression model was calculated for each cereal, which is shown in eqs 5, 6, and 7.
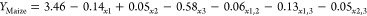
5
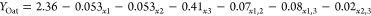
6
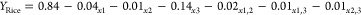
7

**Table 1:** 2^3^ Full Factorial Design (FFD) Including Coded and Natural Variables with the Corresponding Response Value Given as AX in g Per 100 g^a^

	natural value (coded level)			response AX [g/100 g]		
run no.	TFA [M/L] (*X*_1_)	*t* [h] (*X*_2_)	*T* [°C] (*X*_3_)	M01	O01	R01
1	1 (−1)	2 (−1)	100 (−1)	3.82	2.63	0.98
2	3 (1)	2 (−1)	100 (−1)	3.97	2.95	0.95
3	1 (−1)	4 (1)	100 (−1)	4.18	2.83	1.06
4	3 (1)	4 (1)	100 (−1)	4.01	2.63	0.96
5	1 (−1)	2 (−1)	120 (1)	3.06	2.15	0.73
6	3 (1)	2 (−1)	120 (1)	2.59	1.90	0.67
7	1 (−1)	4 (1)	120 (1)	3.13	2.02	0.76
8	3 (1)	4 (1)	120 (1)	2.54	1.73	0.64
9	2 (0)	3 (0)	110 (0)	3.51	2.35	0.83
10	2 (0)	3 (0)	110 (0)	3.59	2.43	0.80
11	2 (0)	3 (0)	110 (0)	3.61	2.33	0.83
12	2 (0)	3 (0)	110 (0)	3.45	2.34	0.83

a AX is calculated as the sum of arabinose and xylose. The values of the monosaccharide analyses are presented in Tables S6–S8.

**Figure 2:**
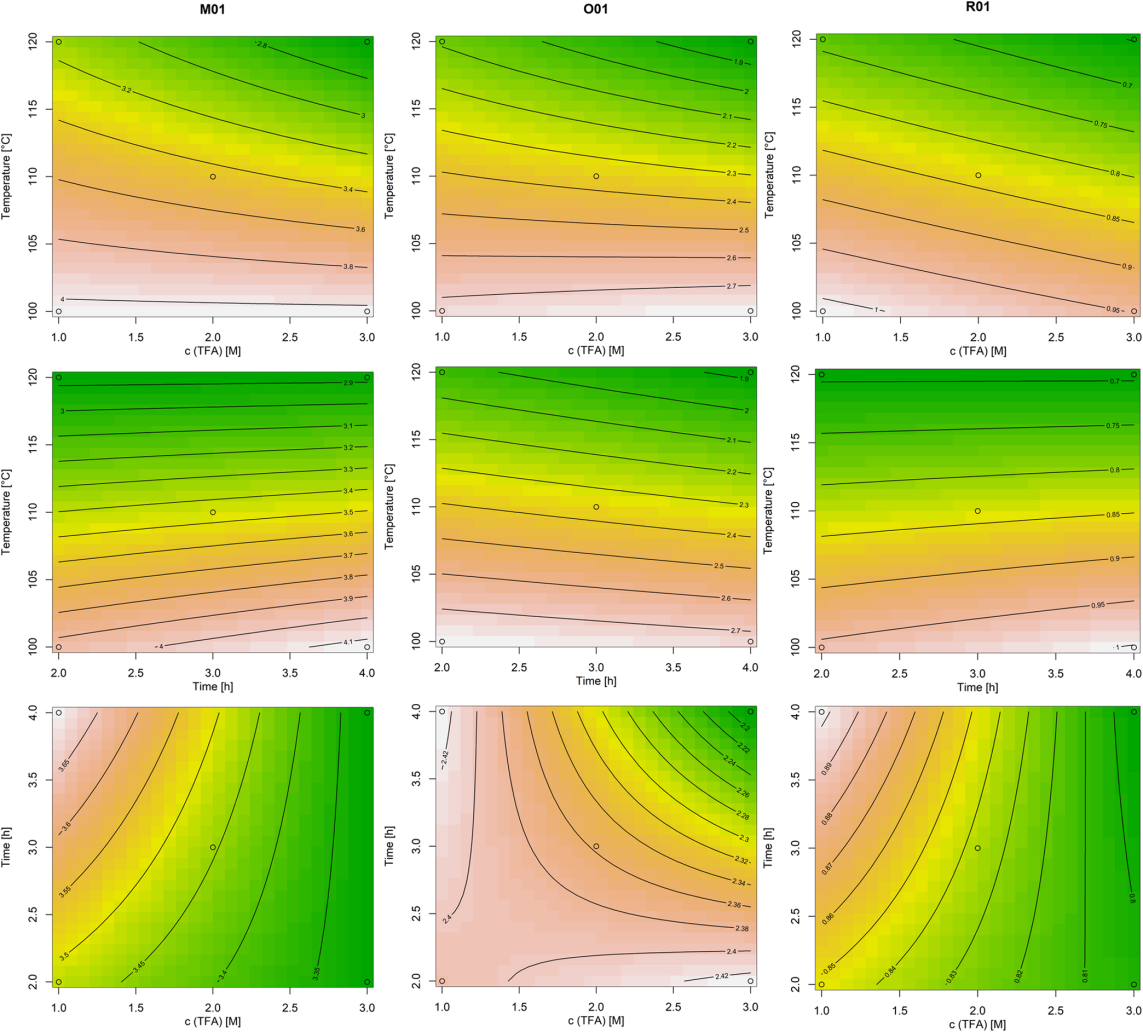
Contour plots of the 2^3^ FFD results, with M01 on the left, O01 in the middle, and R01 on the right. The first row presents the temperature in degrees Celsius on the *y*-axis and the TFA concentration in M on the *x*-axis slice at a hydrolysis time of 3 h. The middle row presents the temperature in degrees Celsius on the *y*-axis and the time in hours on the *x*-axis slice at a concentration of 2 M TFA. The bottom row plots the time in hours on the *y*-axis and the TFA concentration in M on the *x*-axis slice at a temperature of 110 °C. The contour lines indicate the predicted AX content in g per 100 g, while the colors indicate the direction of the steepest ascent to the predicted maximum, from green (= min) over yellow and red to white (= max). The five dots in each plot represent the measurement points of the 2^3^ FFD, as listed in Table 1.

The results of the regression analysis for the FFD are summarized in Table S3. The results showed that across all three raw materials, the temperature influence was highly significant. Neither time nor TFA or any interaction between the factors were significant, which can also be seen in the contour plots in Figure 2. But there was a slight trend indicating a higher yield with decreasing TFA molarity. For all cereals, the predicted path to the optimum AX yield was found at longer hydrolysis times and lower TFA concentrations. Only for oat also shorter times and higher concentrations were predicted to yield higher AX values. However, as the correlations between time and concentration were not significant in all matrices, it was not possible to accurately predict the interaction between these parameters.

A further assessment of the validity of the model was performed using an ANOVA analysis (cf. Table S3). The overall model had moderate *F*-values (24–80) for all grain models, with the first-order terms being the most influential with *F*-values between 64 and 157. The interactions were not significant in any model (*p* < 0.05). It was found that excluding the nonsignificant terms (TWI and time) from the regression equations (cf. eqs 5–7) would have significantly reduced the p-value and thus lead to a considerably poorer fit of the model. Therefore, these terms were kept to ensure that no potentially important effects were prematurely excluded.^28^ Obviously, both the TFA concentration and hydrolysis time exhibited a broad optimum range. The strong statistical performance of the models (*R*^2^ ≥ 0.98, *p* ≤ 0.001) justified its use for further calculations for the path of the steepest ascent.

To identify the maximum response, the path of steepest ascent was followed, as determined by the linear models (cf. eqs 5–7). The step size was uniformly set to 0.5 for the temperature level to conduct experiments at the same time for all three cereals, while the other two variables were adjusted according to the individual regression model. The results of the steepest ascent experiments (performed in duplicate) are summarized in Table 2, and the corresponding monosaccharide values of arabinose, xylose, and galactose are listed in Tables S5–S8 (run nos. 13–24). The AX yields obtained were consistently lower than the predicted values, indicating that the linear model may not have been optimal for accurate prediction. It is hypothesized that this deviation may be explained by the fact that the linear model is too imprecise to reflect the relationships adequately. However, when compared to the results from 2^3^ FFD (cf. Table 1), the yields showed a significant decrease, suggesting that the maximum yield had likely already been achieved in the first factorial experiment. As a result, a CCD was implemented around the 2^3^ FFD to develop a quadratic model as the next step and thus enhance the model prediction fittings.

**Table 2:** Calculations of the Path of the Steepest Ascent, with the Respective Step Size, as Coded Distance and the Corresponding Natural Variables for X1, X2, and X3^a^

			natural value				
run no.	matrix	distance	TFA [M/L] (*X*_1_)	*t* [h] (*X*_2_)	*T* [°C] (*X*_3_)	predicted AX [g/100 g]	obtained AX [g/100 g]
13–14	M01	1.5	2.11	3.29	95	4.37	3.66 ± 0.14
15–16		2	2.32	3.40	90	4.71	2.82 ± 0.01
17–18	O01	1.5	2.28	2.86	95	2.99	2.24 ± 0.01
19–20		2	2.56	2.79	90	3.24	2.02 ± 0.01
21–22	R01	1.5	1.64	3.35	95	1.07	0.93 ± 0.02
23–24		2	1.52	3.56	90	1.16	0.63 ± 0.01

a The predicted AX values in g/100 g, along with the obtained AX value ± SD (*n* = 2), are provided. AX values are calculated as the sum of arabinose and xylose. The monosaccharide analysis values are presented in Tables S6–S8, in accordance with the given run number.

#### 3.2.2 Central Composite Design (CCD)

Since the optimum was already covered within the 2^3^ FFD, as evidenced by the outcomes of the steepest ascent trials, the experimental design was expanded to a CCD to obtain a more reliable prediction of the hydrolysis parameters for maximum AX yield. The CCD trials, with the central point measurements obtained by 3-fold determination (run nos. 31–33), along with the single determined six axial (run nos. 25–30) and eight factorial point (run nos. 1–8) measurements, with the corresponding response (AX yield), are presented in Table 3. The corresponding values of arabinose, xylose, and galactose are listed in Tables S6–S8 (run nos. 1–8 and 25–33). The full second-order polynomial models were generated utilizing the regression of the respective experimental data, which are outlined in eqs 8–10.
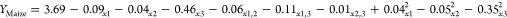
8
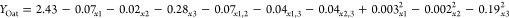
9
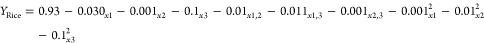
10

**Table 3:** Central Composite Design (CCD) Including Coded and Natural Variables with the Corresponding Response Value, Represented as AX in g Per 100 g (Calculated as the Sum of Arabinose and Xylose)^a^

	natural value (coded level)			response AX [g/100 g]		
run no.	TFA [M/L] (*X*_1_)	*t* [h] (*X*_2_)	*T* [°C] (*X*_3_)	M01	O01	R01
1–8	cf. Table 1					
25	0.27 (−1.73)	3 (0)	110 (0)	3.71	2.43	0.93
26	3.73 (1.73)	3 (0)	110 (0)	3.68	2.17	0.86
27	2 (0)	1.27 (−1.73)	110 (0)	3.68	2.28	0.90
28	2 (0)	4.73 (1.73)	110 (0)	3.22	2.30	0.85
29	2 (0)	3 (0)	93 (−1.73)	3.55	2.37	0.74
30	2 (0)	3 (0)	127 (1.73)	2.07	1.53	0.54
31	2 (0)	3 (0)	110 (0)	3.67	2.44	0.81
32	2 (0)	3 (0)	110 (0)	3.50	2.47	0.98
33	2 (0)	3 (0)	110 (0)	3.71	2.35	0.92

a The values of the monosaccharide analyses are presented in Tables S6–S8, organized according to the run number.

**Figure 3:**
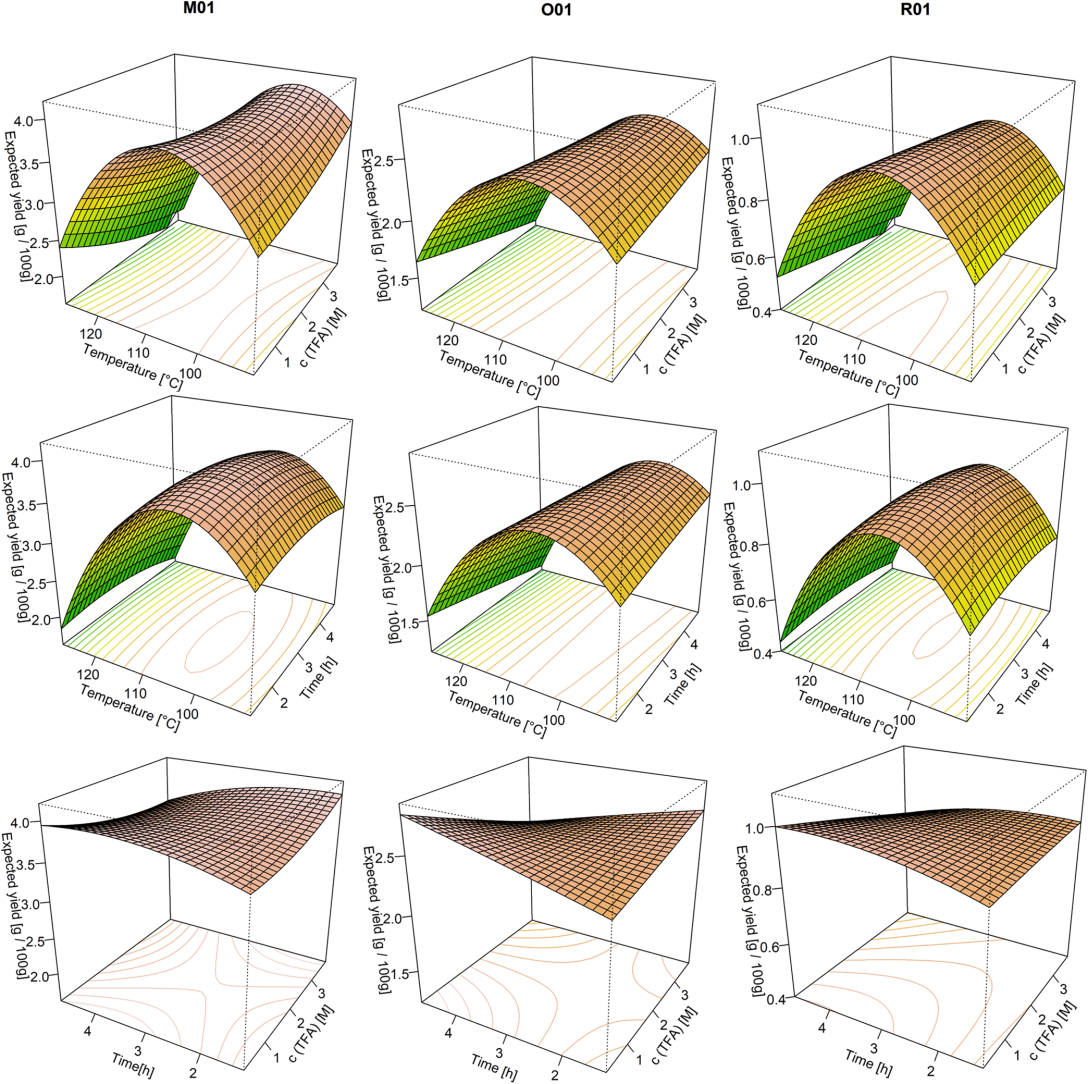
Response surface plots, including contour plots on the bottom, of the CCD results, with M01 on the left, O01 in the middle, and R01 on the right. The first row presents the temperature in degrees Celsius on the *x*-axis and the TFA concentration in M on the *z*-axis slice at a hydrolysis time of 2.4 h. The middle row presents the temperature in degrees Celsius on the *x*-axis and the time in hours on the *z*-axis slice at a concentration of 2 M TFA. The bottom row plots the time in hours on the *x*-axis and the TFA concentration in M on the *z*-axis slice at a temperature of 103 °C. The *y*-axis presents always the expected AX content in g per 100 g, while the colors indicate the direction to the predicted maximum, from green (= min) over yellow and red to white (= max).

The results of the regression analysis of the CCD are in accordance with the FFD the variables of the temperature and the square can be identified as significant (*p* < 0.001) for all three matrices (cf. Table S4). Further analysis of the model identified only the FO and the PQ as significant. The interaction part displayed an *F*-value of <1 for all three models, indicating a significant random error associated with this term. However, the overall models exhibited *p*-values of 0.03–0.003 and an *R*^2^ of approximately 0.8–0.9 as well as no lack of fit (*p* > 0.05) for all three matrices. This indicates that the yield could be modeled with a reasonable degree of accuracy based on the regression models displayed in eqs 8–10. The provided response surfaces in Figure 3 clearly demonstrate the impact of the hydrolysis conditions, as elucidated by statistical analysis. In accordance with the FFD results, the plots in the upper and middle rows collectively confirm the low effect of time and TFA concentration, while the temperature exhibited a pronounced optimum at 100–110 °C. The bottom row, in which the effects of TFA concentration and hydrolysis time are modeled at a constant temperature, demonstrates that only minor effects on the yield can be predicted. The parameters of time and TFA concentration can therefore be regarded as being variable and show a broad maximum within the defined design space, given that they do not have a significant influence on the outcome. Consequently, the model for calculating the optimum yield had to be reduced to the significant factor of temperature, leading to regression eqs 11–13.

The application of these equations to all matrices enabled the identification of an optimal hydrolysis temperature of 103 °C. The findings in this work demonstrate that a change of merely 10 °C in temperature can result in an underestimation of 20–30% in the AX content. This emphasizes the necessity for the establishment of validated methods in order to ensure comparability of data. Furthermore, the observed outcomes were largely consistent across all matrices, demonstrating that the hydrolysis process is suitable for a broad range of cereals, differing in composition and AX structures. These findings are in accordance with those of e.g., Beckendorff et al., who discovered that matrix influences on carbohydrate hydrolysis were absent in corn cob and beech wood xylans.^16^ Thus, it can be concluded that one unified method is effective in releasing the highest levels of arabinose and xylose from AXs in maize, rice, and oat, and further hypothesized that this method is suitable for a broad range of cereals.
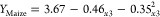
11
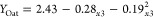
12
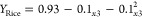
13

#### 3.2.3 Experimental Verification of the Model

For the final verification of the model, the temperature optimum of 103 °C (cf. eqs 11–13) was used and uniform conditions for TFA concentration and hydrolysis time were selected to be situated in the central region of the design space, at a concentration of 2 M TFA and a hydrolysis time of 2.4 h. The predicted AX yields, according to the model calculations and the obtained values from triplicate measurements, are summarized in Table 4 (run nos. 34–42). The CV of all three regression models showed a deviation of less than 10% between the predicted and obtained AX content and beyond that, the values obtained were also the highest values during the entire optimization trials, demonstrating that the model and the maximum yield for these hydrolysis conditions were a good fit. Furthermore, the A/X ratio is an important reference value. This ratio varies by grain type and can provide insights into the structural characteristics, which makes the A/X ratio a valuable marker for refining the quantification accuracy of AX via acid hydrolysis.^34^ The A/X ratios (cf. Tables S6–S8) are decreasing at the proposed optimum for each cereal during the optimization trials. Since arabinose is released faster than xylose, as it is cleaved from side chains attached to the xylose backbone, a low A/X ratio suggests that xylose has been fully released, indicating effective hydrolysis for accurate AX quantification.^14^ In addition, the observed A/X ratios (maize 0.8, oat 0.7, and rice 1.0), are in agreement with previous findings.^2,3,35^

**Table 4:** Parameters for the Verification of the Model, Which Shows the Hydrolysis Parameters, the Predicted and Obtained AX Content in g/100 g, and Their Respective Coefficient of Variation (CV) in Percent^a^

		natural value					
run no.	matrix	TFA [M/L] (*X*_1_)	*t* [h] (*X*_2_)	*T* [°C] (*X*_3_)	predicted AX [g/100 g]	obtained AX [g/100 g]	CV [%]
34–36	M01	2	2.4	103	3.85	4.02 ± 0.12	4.4
37–39	O01				2.54	2.79 ± 0.01	9.8
40–42	R01				0.95	0.99 ± 0.02	4.2

a The values of the monosaccharide analyses are presented in Tables S6–S8, in accordance with the run number. (Calculated as the Sum of Arabinose and Xylose.)

To further verify the release of monosaccharides from AX at the calculated optimum conditions, the HPAEC-PAD chromatograms of M01, O01, and RO1 were analyzed for the presence of residual AX oligomers (AXOs), and the results are shown in Figure S4. For this, the spectra of incomplete hydrolysis (95 °C, 3 h, 2 M TFA) of P-WAXYM were first used to identify the peaks of most prominent AX oligomers (AXO-1 to -3), which were shown to be undetectable under the found optimal hydrolysis conditions for P-WAXYM. The chromatographic conditions were comparable to those applied for AXO identification by Alyassin et al. and should therefore allow the identification of the predominant AXOs with a degree of polymerization of 2 to 8.^36^ The GF-free starch matrix (without AX) was also hydrolyzed under the optimum conditions to identify peaks originating from starch oligomers (SO). The chromatograms of M01, O01, and R01 were evaluated by comparing the retention times with the presence of AXOs and SOs. As can be seen in Figure S4, the majority and predominant peaks that appeared were assigned to starch oligomers. However, for O01 a small signal of AXO-3 and a higher peak area of SO-04 (compared to the starch matrix) was visible. Other small additional peaks at min 20.5 (a), min 29 (b), and min 38.8 (c) can be found in M01, O01, and R01, at min 40.5 (d) in M01 and O1 and at min 37 (e) in R01. These peaks were not found in P-WAXYM or in the starch matrix and could be other SOXs, AXOs, or other oligomeric components, such as those from arabinogalactan. However, these peaks were present at very low levels, and the predominant peaks were all from starch degradation products. These results confirm that under optimum conditions, a sufficient and high release of AX to monosaccharides is carried out in all three matrices, thus verifying predictions from the model.

### 3.3 Validation

First, the selectivity of the instrument was confirmed through measurements of different saccharide standards at a concentration of 0.5 mg/L. The chromatogram of the tested mono- and disaccharide calibration standards is given in Figure S2 with the chromatographic data listed in Table S5. The baseline was stable over the separation time within the first 12 min. Only mannose and xylose showed an incomplete baseline separation with a low-resolution value of 1.2. However, since only low contents of mannose are in the selected cereals, no further optimization was necessary.^37^ The asymmetry values of the peaks were within an acceptable range, demonstrating that the level of selectivity met the criteria for the analysis.

As in carbohydrate analyses via HPAEC-PAD, a classical linear model often does not provide adequate fit, various models, e.g., linear, quadratic, or polynomial, were tested.^38^ A quadratic regression model, using six external calibration points (0.1–20 mg/L), proved to be the best fit, which is consistent with findings in the literature.^38,39^ The calibration curve and the corresponding residual plots are shown in the Figure S3. Both arabinose and xylose achieved an *R*^2^ > 0.995 with the quadratic regression model, meeting the method’s linearity criteria and exhibiting a working range of 0.1–20 mg/L (cf. Table 5).^31^

**Table 5:** Validation Results with Hydrolysis Conditions of 2 M TFA for 2.4 h at 103 °C^a^

validation criteria	sample	result
Linearity^1^	Arabinose	*y* = −0.0342*x*^2^ + 1.7099*x* + 0.2581
		*R*^2^ = 0.998
	Xylose	*y* = −0.00748*x*^2^ + 2.9885*x* + 0.5469
		*R*^2^ = 0.996
LOD^3^	GF wheat starch matrix with 1% P-WAXYH	Arabinose: 94 mg/kg
		Xylose: 248 mg/kg
LOQ^3^	GF wheat starch matrix with 1% P-WAXYH	Arabinose: 313 mg/kg
		Xylose: 827 mg/kg
Measurement precision^3,^^b^	M01	3.95 ± 0.21 g/100 g
	O01	2.67 ± 0.07 g/100 g
	R01	0.95 ± 0.02 g/100 g
Interday precision^1,^^b^	M01	3.73 ± 0.15 g/100 g
	O01	2.56 ± 0.11 g/100 g
	R01	0.94 ± 0.08 g/100 g
Recovery^2,^^b^	GF wheat starch matrix with 1% P-WAXYH	99.4 ± 1.3%
	GF wheat starch matrix with 5% P-WAXYH	91.0 ± 2.2%
	GF wheat starch matrix with 10% P-WAXYH	92.6 ± 1.0%
Trueness^1^	Xylose	98.7 ± 6.3%
	Arabinose	106.8 ± 2.5%
	Galactose	99.9 ± 3.8%
	P-WAXYM*	102.4 ± 0.6%
	P-WAXYH*	97.7 ± 3.6%

a Listed are the validation criteria of the linearity, limit of detection (LOD) and limit of quantification (LOQ), measurement- and interday precision, recovery, and trueness. The linearity result of arabinose and xylose is given as quadratic regression equation with the corresponding *R*^2^. LOD and LOQ values are expressed in mg/kg referring to the whole method, including sample preparation steps. Precision results are given as AX content as mean ± SD. Recovery and trueness results are given as percent recovery of the spiked material ± SD. Number of measurements: ^1^*n* = 3, ^2^*n* = 4, ^3^*n* = 10.

b Calculated as AX = 0.88 * (%xylose + %arabinose) according to Houben and de Ruijter.^24^

HPAEC-PAD is known for its high sensitivity, typically yielding LOD and LOQ values in the μg/L range, e.g., Alyassin et al. reported an LOQ for xylose of 40 μg/L and for arabinose of 30 μg/L.^36^ However, these values pertain to standard monosaccharide dilutions and do not account for the complexities of the sample matrices and complex sample preparation steps. To ensure method reliability, the LOD and LOQ were determined for the entire procedure using a starch-based matrix with low AX content, which is in accordance with the Eurachem Validation Guidelines.^31^ Starch was chosen due to the lack of analyte-free matrices and because high glucose levels mainly limit xylose detection, while other components, such as fats and proteins, were shown to not significantly influence the extraction process.^37^ The LOD and LOQ values of the whole method are in a mg/L range (cf. Table 5). Considering the required 50-fold dilution during sample preparation, AX contents as low as 0.1% in GF grains can be reliably quantified, demonstrating the method’s suitability for sensitive ingredient analysis. It should be noted, however, that the LOD and LOQ values of an HPAEC-PAD method are always dependent on the fitness of the gold electrode.^36^

Precision was evaluated through both intraday and interday assessments. The mean values ± standard deviation for a total of 19 measurements for each cereal are shown in Table 5. For measurement precision, the CV was 1.5% for rice, 2.4% for oat, and 4.6% for maize. In terms of interday precision, the CV was 3.5% for maize, 3.8% for oat, and 7.1% for rice. However, an in-house spiked sample matrix can cause errors, e.g., due to inhomogeneous analyte distribution. Thus, a material-induced uncertainty should be recognized. Nevertheless, all relative deviations in these precision experiments were less than 10%, indicating an acceptable range for the analysis of ingredient analysis.

The overall recovery (cf. Table 5) ranged from 91.0 to 99.4%, with a narrow CV, thereby indicating that the recovery met the analytical requirements. In the absence of CRMs, a practical approach was adopted to assess the trueness. This approach included two main steps: first, evaluating the release of monosaccharides of P-WAXYH and P-WAXYM, a high- and medium-viscosity wheat AX, and second, assessing the degradation of monosaccharide standards, to determine any loss of free monosaccharides during sample preparation. The optimization process revealed no significant matrix differences among GF cereals, suggesting that the wheat AX used was suitable for monitoring the release of arabinose and xylose in the process. Overall, the detected values for arabinose and xylose were 102.4 ± 0.6 and 97.7 ± 3.6% for P-WAXYM and P-WAXY-H, respectively, and 98.7 ± 6.3 and 106.8 ± 2.5% for xylose and arabinose, respectively, indicating nearly complete recovery (cf. Table 5). As previously outlined by Gao et al., these findings underscore that TFA is advantageous for acid hydrolysis of hemicelluloses, as it is less destructive in this setting for xylose and arabinose.^15,40^ The used AX sources for recovery experiments are already isolated materials that exhibit modifications and a low degree of cross-linking. Consequently, the observed hydrolysis efficiency may differ from that of the real samples, thereby introducing potential deviations. However, the accuracy can only be verified by using certified materials with well-defined AX amounts. As these materials are currently not available, the applied approach and the derived outcomes are adequate for confirming the trueness of the method.

The hydrolysis effectively releases the maximum amount of arabinose and xylose from the tested grains. However, these monosaccharides can originate not only from AX but also in smaller amounts from other carbohydrates, such as arabinogalactans. To address the lack of selectivity, Courtin & Delcour proposed an adjustment using the factor subtracting “galactose-0.7” from the arabinose and xylose content.^25^ This study confirmed galactose stability for this hydrolysis (*cf.*Table 5), enabling the correction of arabinose from e.g., arabinogalactan using appropriate correction factors. However, selectivity must be considered in a variety-specific context, since the amounts vary in cereal varieties. Establishing cereal-specific correction factors requires enormous data regarding the detailed composition of dietary fiber ingredients, which would allow further improvement. After this, the method selectivity uncertainty can be determined, and the overall method uncertainty needs to be addressed through, e.g., interlaboratory comparisons. Nevertheless, this method approach has the potential to be developed to integrate AX determination into a variety testing as routine constituent analysis.

Overall, the analysis of AXs in cereals is challenging due to the lack of a standardized methodology and inconsistency hydrolysis conditions for monosaccharide determination, affecting the reliability and comparability of studies. This study investigated acid hydrolysis conditions for three diverse GF cereals: maize, oat, and rice, differing notably in their matrix and AX composition. The acid hydrolysis was optimized in regard to hydrolysis time, TFA concentration, and temperature based on DoE, while monosaccharide detection was conducted using HPAEC-PAD. The regression model was validated for all three matrices, demonstrating a deviation of less than 10% CV in the fit of the model for the prediction of the AX content. Temperature exerts a considerable influence on the AX yield and A/X ratio, whereas time and acid concentration can be varied over a broader range with only minimal impact. Different matrices showed minor effects on hydrolysis, which underlines the applicability of a unified method. The optimal hydrolysis conditions (103 °C, 2.4 h, 2 M TFA) were finally validated. However, the absence of suitable CRMs renders the validation of the accuracy a challenging undertaking. Although this work represents a significant advance in the implementation of a standardized method for the determination of AX in cereals, the development of CRMs as well as the identification of cereal-specific calculation factors are essential requirements for providing a basis for the comparison and trustworthiness of analytical methods.

## Supporting Information Available

Chromatographic gradient conditions for HPAEC-PAD analysis (Table S1); composition values of the samples M01, R01, and O01 (Table S2); statistical evaluation of the FFD (Table S3); statistical evaluation of the CCD (Table S4); HPAEC-PAD method resolution and separation parameters (Table S5); monosaccharide analysis results for maize (M01) (Table S6); monosaccharide analysis results for oat (O01) (Table S7); monosaccharide analysis results for rice (R01) (Table S8); amino acid composition of M01, O01, and R01 (Figure S1); HPAEC-PAD chromatogram of a PA20-Fast Column separation for the calibration standards (Figure S2); quadratic regression of HPAEC-PAD response and corresponding residual plots (Figure S3); and HPAEC-PAD chromatograms of occurring AX and starch oligomers from incomplete hydrolysis compared to the occurrence at the optimum hydrolysis level (Figure S4) (PDF)

## Notes

**Declaration of Generative AI and AI-Assisted Technologies in the Writing Process**: To enhance the readability of this work, the authors employed the use of DeepL Write. The content was subsequently reviewed and edited as necessary, and the authors assume full responsibility for the integrity of the published material.

## Abbreviations

AX: Arabinoxylan
A/X: Arabinose to xylose
AXO: Arabinoxylan oligomer
CCD: Central composite design
CRM: Certified reference material
CV: Coefficient of variation
DM: Dry matter
DoE: Design of Experiments
FA: Ferulic acid
FFD: Full factorial design
FO: First order
GF: Gluten-free
HPAEC-PAD: High-performance anion exchange chromatography with pulsed amperometric detection
IC: Ion chromatography
LOD: Limit of detection
LOQ: Limit of quantification
P-WAXYH: Wheat arabinoxylan high viscosity
P-WAXYM, PQ: Quadratic part; Wheat arabinoxylan medium viscosity
RM: Reference material
RSM: Response surface methodology
SCFA: Short-chain fatty acid
SD: Standard deviation
SO: Starch oligomer
TDF: Total dietary fiber
TFA: Trifluoracetic acid
TPC: Total phenolic content
TWI: Two-way-interaction
PQ: quadratic term
UHQ water: Ultrapure water
