# Supplementary material for: Optimization and Validation of Arabinoxylan Quantification in Gluten-Free Cereals via HPAEC-PAD Based on Design of Experiments
Source: J Agric Food Chem. 2025 Apr 2;73(15):9309–19. doi: 10.1021/acs.jafc.5c02445 (PMC12007097; doi:10.1021/acs.jafc.5c02445)
Supplement: Supplementary file 1 — jf5c02445_si_001.pdf [file jf5c02445_si_001.pdf]

SUPPLEMENTARY DATA

Optimization and Validation of Arabinoxylan Quantification in Gluten-Free Cereals via HPAEC-PAD Based on Design of Experiments

Katharina Hoefler [†,‡], Ulrich Sukop[‡], Stefan Scheler[‖], Elisabeth Reiter[†], Denisse Bender[‡], Mario Jekle[§], Regine Schoenlechner[‡*], Stefano D'Amico[†]

[†] AGES – Austrian Agency for Health and Food Safety, Institute for Animal Nutrition and Feed, Spargelfeldstraße 191, 1220 Vienna, Austria

[‡] BOKU – University, Department of Biotechnology and Food Science, Muthgasse 18, 1190 Vienna, Austria

[§] University of Hohenheim – Department of Plant-Based Foods, Garbenstraße 25, 70599 Stuttgart, Germany

[‖] University of Applied Sciences Kaiserslautern – Department of Applied Logistics and Polymer Sciences, Carl-Schurz-Straße 10 – 16, 66953 Pirmasens, Germany

*Corresponding author: Regine.Schoenlechner@boku.ac.at

Table S1: Chromatographic gradient conditions for monosaccharide analysis by HPAEC-PAD at a constant flow rate of 0.2 mL / min with a PA20-Fast Column (2 x 100 mm, 4 µm) at 30 °C.

**Time (min)**	**%B (200 mM NaOH)**	**%C (1000 mM NaOH)**
0	1.0	0
7.5	1.0	0
12.5	7.0	0
22.5	17.0	0
32.5	100.0	0
32.6	0	20
37.5	30	70
40.0	30.0	70
40.1	1.0	0
55.0	1.0	0

Table S2: Composition values of the three matrices M01, O01 and R01 according to **Figure 1**. Values are given as mean ± SD (n=3) in dry matter.

**Component**	**M01**	**O01**	**R01**
Starch [g/100g]	72.6 ± 1.0	62.3 ± 2.9	81.7 ± 6.8
Fat [g/100g]	4.18 ± 0.45	5.94 ± 0.08	2.74 ± 0.21
Protein [g/100g]	6.77 ± 0.09	15.2 ± 0.2	9.67 ± 0.03
TDF [g/100g]	10.0 ± 0.5	11.3 ± 0.4	4.48 ± 0.82
Ash [g/100g]	1.26 ± 0.02	2.56 ± 0.21	1.44 ± 0.01
TPC [mg FA/g]	4.25 ± 0.22	1.23 ± 0.02	0.71 ± 0.04
- bound	3.71 ± 0.15	0.908 ± 0.113	0.667 ± 0.120
- conjugated	0.307 ± 0.016	0.206 ± 0.009	0.009 ± 0.003
- free	0.238 ± 0.039	0.120 ± 0.009	0.030 ± 0.010

Table S3: Statistical evaluation of the mathematical equations 5 – 7, derived from the 2^3 FFD. The data set includes the t-test results of the FFD coefficients and ANOVA of the entire model, comprising first-order (FO) and two-way interaction (TWI) terms, in addition to the lack of fit and R^2. The following significance codes are attributed to the respective p-values: ns (not significant) ≥ 0.05; * < 0.05; ** < 0.001; *** <0.0001.

**Source**	**M01**			**O01**			**R01**		
**t-test**	**t-value**	**p-value**	**Signif. code**	**t-value**	**p-value**	**Signif. code**	**t-value**	**p-value**	**Signif. code**
Intercept	105.17	$2 * 10^{-9}$	***	97.30	$2 * 10^{-9}$	***	149	$3 * 10^{-10}$	***
X1 (TFA)	-3.36	0.02	*	-1.77	0.14	ns	-5.63	0.00	**
X2 (t)	1.30	0.25	ns	-1.77	0.14	ns	1.64	0.16	ns
X3 (T)	-14.48	$3 * 10^{-5}$	***	-13.65	$4 * 10^{-5}$	***	-20.9	$5 * 10^{-6}$	***
X1:X2	-1.37	0.23	ns	-2.36	0.06	ns	-2.36	0.06	ns
X1:X3	-3.23	0.023	*	-2.78	0.04	*	-0.91	0.41	ns
X2:X3	-1.18	0.29	ns	-0.76	0.48	ns	-1.64	0.16	ns
**ANOVA**	**F-value**	**p-value**	**Signif. code**	**F-value**	**p-value**	**Signif. code**	**F-value**	**p-value**	**Signif. code**
Model	39.4	0.0007	**	34.4	0.0007	**	80.1	$8 * 10^{-5}$	***
FO	74.2	0.0001	**	64.2	0.0002	**	157	$2 * 10^{-5}$	***
TWI	4.57	0.067	ns	4.62	0.07	ns	3.03	0.13	ns
Lack of fit	4.42	0.13	ns	6.92	0.08	ns	2.70	0.21	ns
R^2		0.98			0.98			0.99	

Table S4: Statistical evaluation of the mathematical equations 8 – 10, derived from the CCD. The data set includes the t-test results of the CCD coefficients and ANOVA of the entire model, comprising first-order (FO) and two-way interaction (TWI) and polynomial (PQ) terms, in addition to the lack of fit and R^2. The following significance codes are attributed to the respective p-values: ns (not significant) ≥ 0.05; * < 0.05; ** < 0.001; *** <0.0001.

**Source**	**M01**			**O01**			**R01**		
**t-test**	**t-value**	**p-value**	**Signif. code**	**t-value**	**p-value**	**Signif. code**	**t-value**	**p-value**	**Signif. code**
Intercept	24	$1.8 * 10^{-9}$	***	19	$1.7 * 10^{-8}$	***	19	$1.3 * 10^{-8}$	***
X1 (TFA)	-1.2	0.27	ns	-1.2	0.26	ns	-1.3	0.23	ns
X2 (t)	-0.5	0.61	ns	-0.38	0.71	ns	-0.040	0.96	ns
X3 (T)	-7.0	$6.5 * 10^{-5}$	***	-5.0	0.0007	***	-4.7	0.001	**
X1:X2	-0.61	0.56	ns	-0.81	0.43	ns	-0.48	0.64	ns
X1:X3	-1.16	0.28	ns	-0.56	0.59	ns	-0.38	0.71	ns
X2:X3	-0.10	0.93	ns	-0.48	0.64	ns	-0.049	0.96	ns
$X1^2$	0.47	0.64	ns	-0.049	0.96	ns	-0.039	0.97	ns
$X2^2$	-0.60	0.56	ns	-0.032	0.98	ns	-0.42	0.68	ns
$X3^2$	-5.19	0.0006	***	-3.3	0.009	**	-4.7	0.001	**
**ANOVA**	**F-value**	**p-value**	**Signif. code**	**F-value**	**p-value**	**Signif. code**	**F-value**	**p-value**	**Signif. code**
Model	7.5	0.003	**	3.7	0.03	*	4.5	0.02	*
FO	10.4	0.003	**	6.1	0.02	*	3.8	0.05	ns
TWI	0.5	0.68	ns	0.4	0.77	ns	0.76	0.54	ns
PQ	11.7	0.002	**	4.6	0.03	*	8.9	0.005	**
Lack of fit	8.3	0.11	ns	13.0	0.07	ns	1.4	0.48	ns
R^2		0.88			0.79			0.81	

Table S5: The HPAEC-PAD method resolution and separation parameters for the studied mono- and disaccharides.

Parameter	Fuc	Ara	Gal	Glu	Xyl	Man	Fru	Rib	Mel
Resolution factor	7.9	2.9	2.4	2.8	1.2	1.9	1.6	2.2	NA
Asymmetry	1.37	1.08	1	0.99	NA	NA	0.95	0.9	0.95
Theoretical plates	1667	3603	4086	4442	5264	5486	5970	6078	5699

Fuc = Fucose, Ara = Arabinose, Gal = Galactose, Xyl = Xylose, Man = Mannose, Fru = Fructose, Rib = Ribose, Mel = Melibiose.

Table S6: The following table presents the monosaccharide analysis results for **maize (M01)** along with the corresponding run number in g / 100g. Run number 1 – 12 correspond to the FFD in **Table 1**, run 13 – 24 to the path of the steepest ascent trials according to **Table 2**, run 25 – 33 correspond to the CCD results according to **Table 3** and run 34 – 36 correspond to the verification trials according to **Table 4**. The results are calculated as a sum, in accordance with the methodologies proposed by Houben and de Ruijter, as well as by Courtin and Delcour. Additionally, the arabinose to xylose ratio is presented.

Run No	Arabinose (g/100g)	Xylose (g/100g)	Galactose (g/100g)	AX Sum (g/100g)	AX Houben[1] (g/100g)	AX Courtin[2] (g/100g)	A/X ratio
1	1.74	2.08	0.63	3.82	3.36	2.98	0.84
2	1.78	2.19	0.68	3.97	3.49	3.08	0.81
3	1.93	2.24	0.70	4.18	3.68	3.24	0.86
4	1.85	2.16	0.68	4.01	3.53	3.12	0.86
5	1.50	2.69	0.57	3.06	2.69	1.77	0.96
6	1.24	2.28	0.49	2.59	2.28	1.51	0.92
7	1.53	2.76	0.58	3.13	2.76	1.81	0.96
8	1.28	2.23	0.48	2.54	2.23	1.44	1.02
9	1.66	1.85	0.63	3.51	3.09	2.70	0.90
10	1.65	1.94	0.60	3.59	3.16	2.79	0.85
11	1.65	1.96	0.62	3.61	3.18	2.79	0.84
12	1.65	1.80	0.61	3.45	3.04	2.66	0.91
13	1.73	2.03	0.65	3.76	3.31	2.91	0.85
14	1.58	1.98	0.62	3.56	3.13	2.75	0.80
15	1.18	1.65	0.51	2.83	2.49	2.17	0.71
16	1.15	1.66	0.50	2.81	2.47	2.16	0.69
25	1.81	1.90	0.61	3.71	3.27	2.89	0.95
26	1.83	1.85	0.64	3.68	3.24	2.84	0.99
27	1.82	1.86	0.56	3.68	3.24	2.89	0.98
28	1.61	1.61	0.57	3.22	2.84	2.49	1.00
29	1.77	1.78	0.63	3.55	3.12	2.73	0.99
30	1.15	0.93	0.47	2.07	1.83	1.54	1.24
31	1.82	1.86	0.64	3.67	3.23	2.84	0.98
32	1.69	1.81	0.63	3.50	3.08	2.80	0.98
33	1.83	1.89	0.64	3.71	3.27	2.87	0.97
34	1.81	2.16	0.60	3.97	3.49	3.12	0.84
35	1.91	2.24	0.63	4.16	3.66	3.27	0.85
36	1.82	2.10	0.60	3.93	3.45	3.09	0.87

[1] Calculated according to Houben and de Ruijter with AX = 0.88 * (%xylose + %arabinose) [1].
[2] Calculated according to Courtin and Delcour with AX = 0.88 * [(%arabinose – 0.7 * %galactose) + %xylose] [2].

Table S7: The following table presents the monosaccharide analysis results for **oat (O01)** along with the corresponding run number in g / 100g. Run number 1 – 12 correspond to the FFD in **Table 1**, run 13 – 24 to the path of the steepest ascent trials according to **Table 2**, run 25 – 33 correspond to the CCD results according to **Table 3** and run 37 – 39 correspond to the verification trials according to **Table 4**. The results are calculated as a sum, in accordance with the methodologies proposed by Houben and de Ruijter, as well as by Courtin and Delcour. Additionally, the arabinose to xylose ratio is presented.

Run No	Arabinose (g/100g)	Xylose (g/100g)	Galactose (g/100g)	AX Sum (g/100g)	AX Houben[1] (g/100g)	AX Courtin[2] (g/100g)	A/X ratio
1	1.20	1.43	0.75	2.63	2.32	1.85	0.84
2	1.28	1.67	0.81	2.95	2.60	2.09	0.76
3	1.16	1.57	0.83	2.83	2.49	1.98	0.81
4	1.15	1.48	0.77	2.63	2.32	1.85	0.78
5	1.04	1.89	0.70	2.15	1.89	1.25	0.94
6	0.90	1.67	0.64	1.90	1.67	1.12	0.91
7	0.99	1.78	0.69	2.02	1.78	1.17	0.95
8	0.81	1.52	0.60	1.73	1.52	1.02	0.88
9	1.09	1.27	0.74	2.35	2.07	1.61	0.86
10	1.12	1.31	0.76	2.43	2.14	1.67	0.86
11	1.08	1.25	0.74	2.33	2.05	1.60	0.86
12	1.05	1.30	0.73	2.34	2.06	1.61	0.81
17	0.97	1.27	0.70	2.24	1.97	1.53	0.76
18	0.95	1.30	0.70	2.24	1.98	1.55	0.73
19	0.82	1.20	0.62	2.02	1.78	1.40	0.68
20	0.84	1.19	0.64	2.03	1.79	1.39	0.71
25	1.21	1.22	0.65	2.43	2.14	1.74	1.00
26	1.07	1.10	0.70	2.17	1.91	1.48	0.97
27	1.17	1.11	0.56	2.28	2.00	1.66	1.05
28	1.17	1.13	0.75	2.30	2.02	1.56	1.03
29	1.19	1.18	0.74	2.37	2.09	1.63	1.01
30	0.87	0.66	0.69	1.53	1.34	0.92	1.32
31	1.22	1.22	0.78	2.44	2.14	1.66	1.00
32	1.23	1.23	0.78	2.47	2.17	1.69	1.00
33	1.18	1.18	0.76	2.35	2.07	1.60	1.00
37	1.20	1.59	0.75	2.79	2.46	2.00	0.76
38	1.22	1.56	0.76	2.78	2.45	1.98	0.78
39	1.22	1.58	0.74	2.81	2.47	2.01	0.77

[1] Calculated according to Houben and de Ruijter with AX = 0.88 * (%xylose + %arabinose) [1].
[2] Calculated according to Courtin and Delcour with AX = 0.88 * [(%arabinose – 0.7 * %galactose) + %xylose] [2].

Table S8: The following table presents the monosaccharide analysis results for **rice (R01)** along with the corresponding run number in g / 100g. Run number 1 – 12 correspond to the FFD in **Table 1**, run 13 – 24 to the path of the steepest ascent trials according to **Table 2**, run 25 – 33 correspond to the CCD results according to **Table 3** and run 40 – 42 correspond to the verification trials according to **Table 4**. The results are calculated as a sum, in accordance with the methodologies proposed by Houben and de Ruijter, as well as by Courtin and Delcour. Additionally, the arabinose to xylose ratio is presented.

Run No	Arabinose (g/100g)	Xylose (g/100g)	Galactose (g/100g)	AX Sum (g/100g)	AX Houben[1] (g/100g)	AX Courtin[2] (g/100g)	A/X ratio
1	0.47	0.51	0.15	0.98	0.86	0.77	0.92
2	0.47	0.48	0.17	0.95	0.83	0.73	0.98
3	0.50	0.56	0.19	1.06	0.93	0.82	0.88
4	0.46	0.50	0.18	0.96	0.85	0.74	0.90
5	0.38	0.35	0.15	0.73	0.64	0.41	1.09
6	0.34	0.33	0.14	0.67	0.59	0.38	1.03
7	0.40	0.36	0.16	0.76	0.67	0.42	1.09
8	0.33	0.30	0.14	0.64	0.56	0.36	1.09
9	0.45	0.38	0.17	0.83	0.73	0.63	1.18
10	0.44	0.36	0.17	0.80	0.70	0.60	1.20
11	0.44	0.39	0.17	0.83	0.73	0.62	1.14
12	0.44	0.39	0.17	0.83	0.73	0.63	1.12
21	0.49	0.45	0.19	0.94	0.83	0.71	1.09
22	0.47	0.46	0.18	0.92	0.81	0.70	1.02
23	0.29	0.35	0.12	0.64	0.56	0.49	0.83
24	0.30	0.33	0.14	0.62	0.55	0.47	0.91
25	0.48	0.45	0.14	0.93	0.82	0.73	1.07
26	0.42	0.44	0.16	0.86	0.76	0.66	0.96
27	0.48	0.41	0.12	0.90	0.79	0.71	1.17
28	0.43	0.43	0.16	0.85	0.75	0.66	1.01
29	0.43	0.31	0.15	0.74	0.65	0.56	1.38
30	0.32	0.22	0.15	0.54	0.48	0.39	1.44
31	0.41	0.41	0.17	0.81	0.71	0.70	1.00
32	0.48	0.50	0.18	0.98	0.86	0.75	0.96
33	0.45	0.47	0.17	0.92	0.81	0.70	0.96
40	0.49	0.49	0.17	0.98	0.86	0.76	1.02
41	0.51	0.50	0.18	1.01	0.89	0.77	1.02
42	0.50	0.48	0.18	0.98	0.86	0.75	1.04

[1] Calculated according to Houben and de Ruijter with AX = 0.88 * (%xylose + %arabinose) [1].
[2] Calculated according to Courtin and Delcour with AX = 0.88 * [(%arabinose – 0.7 * %galactose) + %xylose] [2].

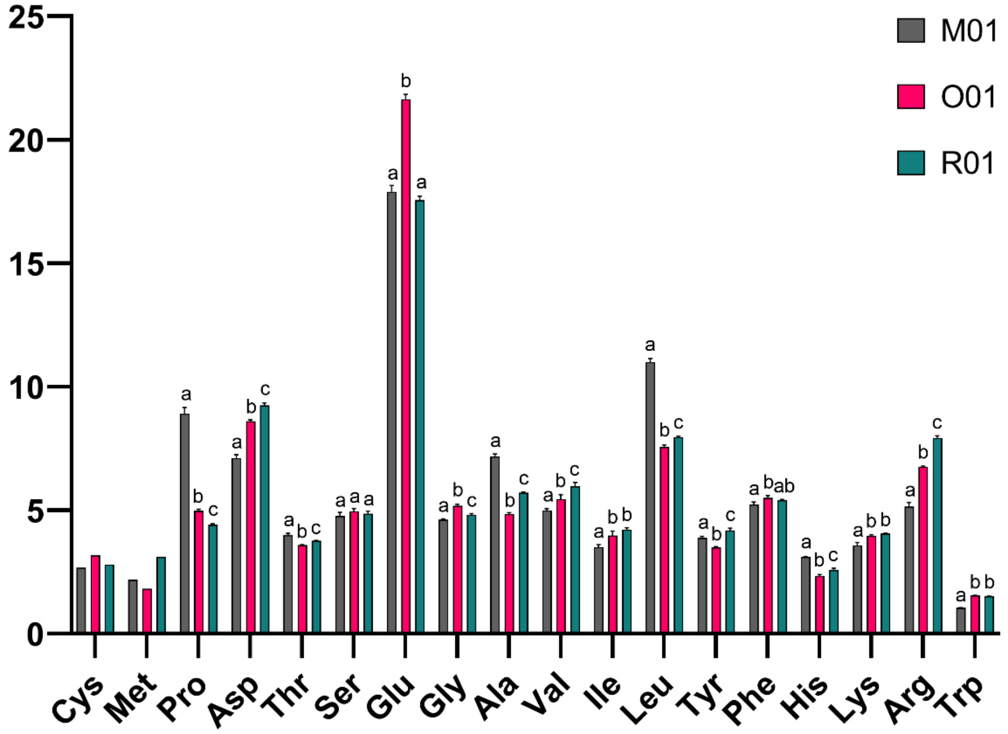

Figure S1: Amino acid composition of M01, O01 and R01. Data are presented as mean + SD in g amino acid / 100 g protein (n = 3 except. Cys, Met (n =1) and Trp (n = 2)) with the low letters indicating significant differences ($p < 0.05$).

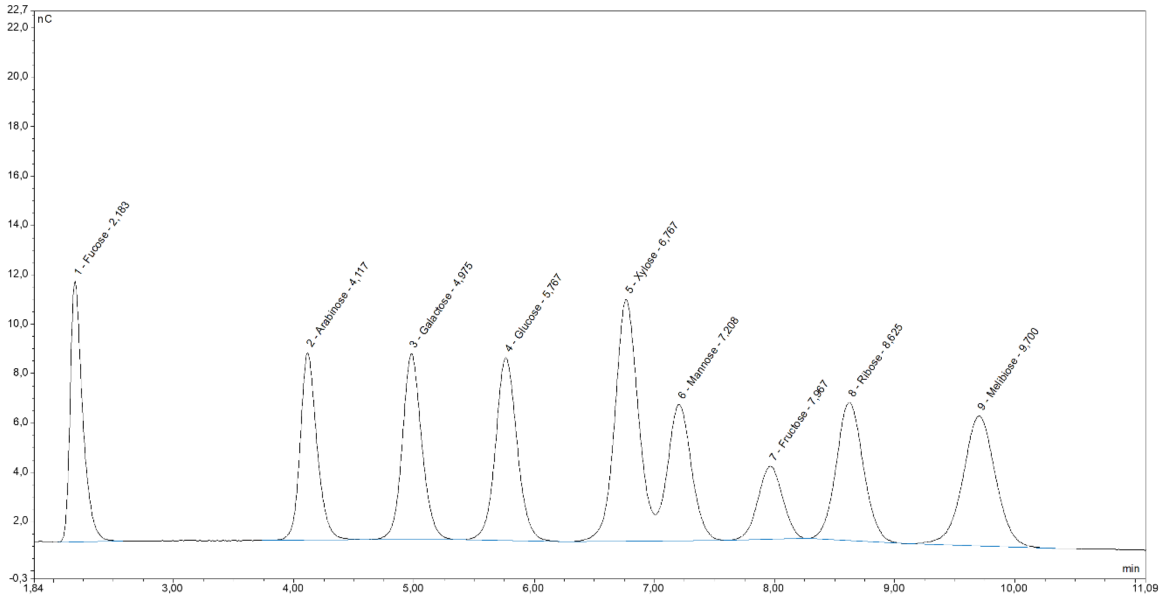

Figure S2: HPAEC-PAD chromatogram of a PA20-Fast Column separation for the calibration standards at a concentration of 0.5 mg/L with the corresponding retention times.

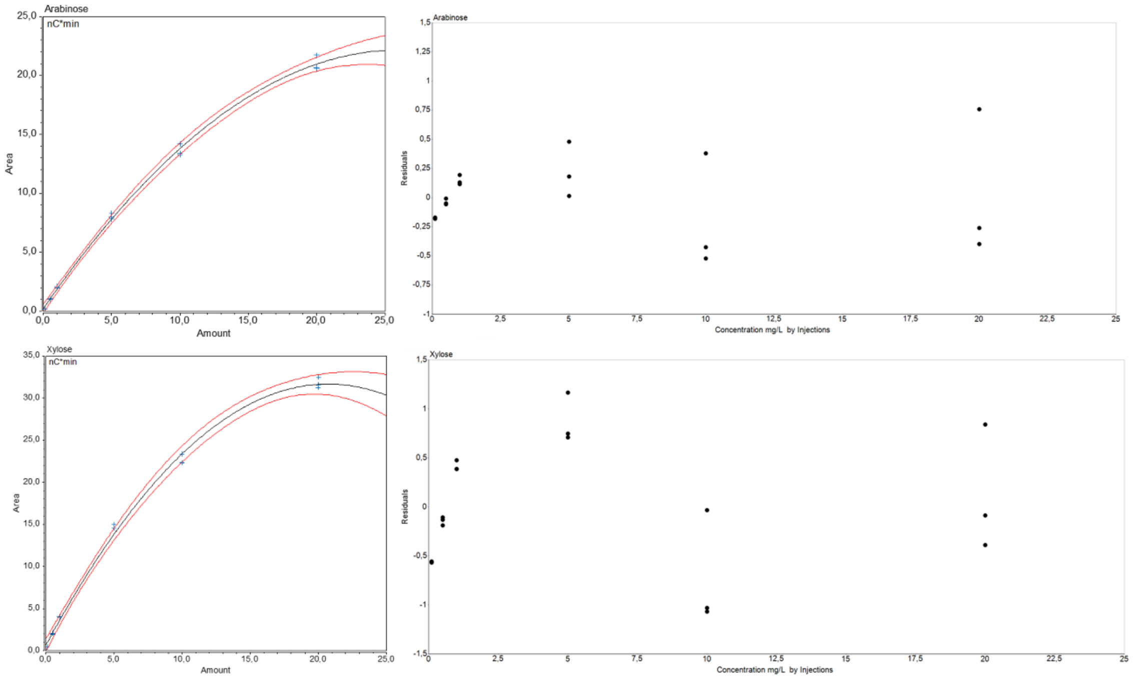

Figure S3: Quadratic regression of HPAEC-PAD response for arabinose (top) and xylose (bottom) for six calibration standards (0.1 – 20 ppm) on the left, with the red line indicating the 95 % confidence interval (n = 3). The corresponding residual plots are given on the right side, respectively.

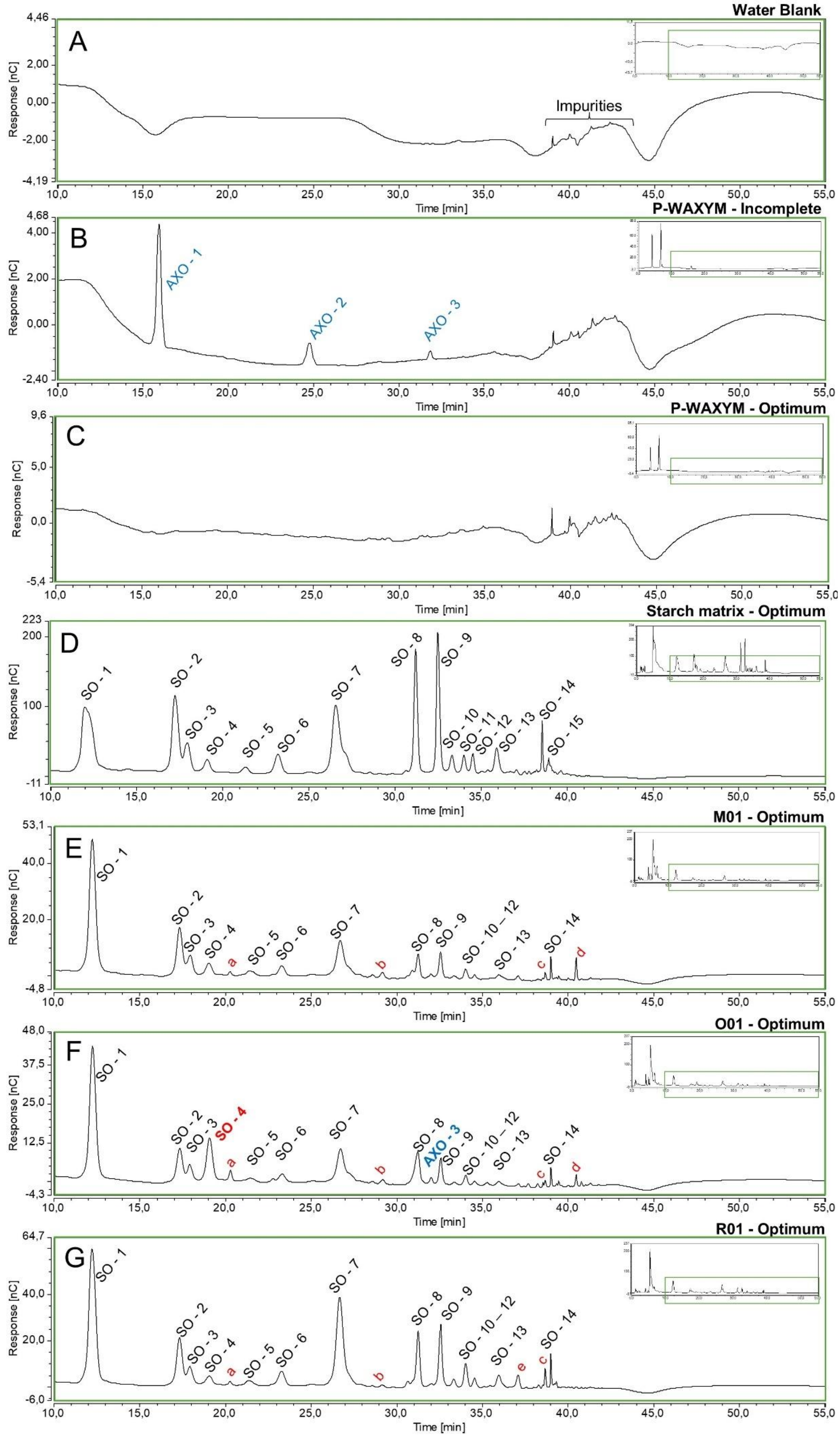
Water Blank
A
Impurities
P-WAXYM - Incomplete
B
AXO - 1
AXO - 2
AXO - 3
P-WAXYM - Optimum
C
Starch matrix - Optimum
D
SO - 1
SO - 2
SO - 3
SO - 4
SO - 5
SO - 6
SO - 7
SO - 8
SO - 9
SO - 10
SO - 11
SO - 12
SO - 13
SO - 14
SO - 15
M01 - Optimum
E
SO - 10 _ 12
a
b
c
d
O01 - Optimum
F
AXO - 3
R01 - Optimum
G
e
Response [nC]
Time [min]

86

Figure S4: Exemplary HPAEC-PAD chromatograms of a PA20-Fast column separation for the alignment of occurring oligomers in the time range of 10 – 55 min with the chromatographic conditions given in Table S1. With AXO (labelled in blue) = arabinoxylan oligomer, SO (labelled in black) = starch oligomer, both numbered in the order of appearance and in lower case letters a – e (labelled in red) unknown peaks in the samples in the order of appearance. The chromatograms (from top to bottom) are assigned in capital letters with A = water blank, showing impurities in the range of min 39 – 44, with B = P-WAXYM as incomplete hydrolysed AX standard (95 °C, 3 h, 2 M TFA), where AXO were identified, with C = P-WAXYM at the optimum of the hydrolysis conditions (103 °C, 2.4 h, 2 M TFA), whereby no AXO can be identified, with D = starch matrix at the optimum, whereby SO - 1 to - 15 are identified as oligomers, which are to be expected due to the starch matrix in the samples and with E = M01, F = O01 and G = R01, which were each hydrolysed at the optimum and AXO and SO are assigned on the basis of the retention times.

References

(1) Houben, R.; Ruijter, C. F. de; Brunt, K. Determination of the Pentosan Content of Wheat Products by Hydrolysis, Glucose Oxidase Treatment and Analysis by HPAEC/PAD, *Journal of Cereal Science.* **1997**, *26,* pp. 37–46.

(2) Courtin, C. M.; Delcour, J. A. Physicochemical and Bread-Making Properties of Low Molecular Weight Wheat-Derived Arabinoxylans, *Journal of Agricultural and Food Chemistry.* **1998**, *46,* pp. 4066–4073.
